# Predicting speech-in-noise ability in normal and impaired hearing based on auditory cognitive measures

**DOI:** 10.3389/fnins.2023.1077344

**Published:** 2023-02-07

**Authors:** Timothy D. Griffiths

**Affiliations:** Biosciences Institute, Newcastle University Medical School, Newcastle upon Tyne, United Kingdom

**Keywords:** auditory cognition, speech in noise, behaviour, cortex, brain

## Abstract

Problems with speech-in-noise (SiN) perception are extremely common in hearing loss. Clinical tests have generally been based on measurement of SiN. My group has developed an approach to SiN based on the auditory cognitive mechanisms that subserve this, that might be relevant to speakers of any language. I describe how well these predict SiN, the brain systems for them, and tests of auditory cognition based on them that might be used to characterise SiN deficits in the clinic.

## Introduction

The ability to hear speech in noisy listening situations is the most important aspect of natural listening carried out by humans. Problems with speech-in-noise (SiN) are ubiquitous in peripheral hearing loss due to cochlear damage, and also in common brain disorders including stroke and dementia. SiN ability is dependent on cochlear function and can be predicted to an extent by the audiogram, but also depends on cortical analysis: even for aspects of auditory pattern analysis that are independent of language.

From first principles, SiN might depend on mechanisms that allow separation of foreground from background elements, the grouping together of foreground elements over time, selective attention to these, and linguistic analysis. I focus here on auditory cognitive mechanism that are responsible for the first two processes. This represents an effort to characterise mechanisms beyond the cochlea for the detection of sound that might account for the large variance in SiN ability that is not due to the audiogram. I do not dismiss the importance and relevance of linguistic factors: the aim of the exercise is to define generic brain mechanisms relevant to speakers of any language of any ability. The data suggest a large amount of the variance can be defined in this way. I will describe behavioural measures of auditory cognition that predict SiN ability and the brain basis for these.

Clinically, behavioural and brain measures of this level of auditory cognition provide a potential means to characterise cortical mechanisms for auditory cognition that explain variation in the SiN listening that is not accounted for by the audiogram. Such measures have potential use in the prediction of hearing outcome after restoration by hearing aids and cochlear implantation. They will not replace SiN tests clinically but suggest a means to partition the causes of SiN impairment that might guide intervention and rehabilitation.

## Auditory cognitive mechanisms for speech-in-noise analysis

**FIGURE 1:**
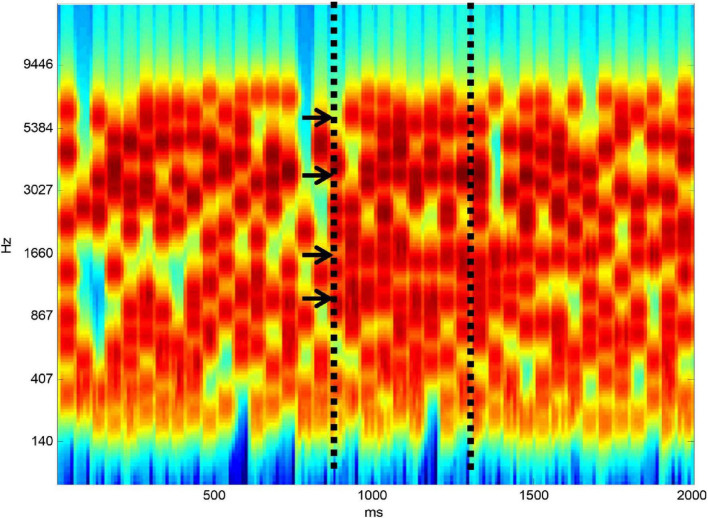
Figure-ground stimulus. The “ground” stimulus contains random elements distributed in frequency-time space. At a certain point in time, shown by the first vertical dashed line, a certain number of elements (four here–shown by arrows) are constrained to remain constant from one time frame to the next. If there are enough elements and they are on for long enough a “figure” emerges perceptually from the background.

Listening to speech in noise is complicated even at the level of auditory analysis before linguistic processing. Speech is a complex broadband signal that contains features in frequency-time space that change over time. This must be separated from background noise that overlaps in frequency and in time. Figure 1 shows a stimulus developed by my group to define the ability of subjects to carry out the figure-ground separation that is required at the initial stage of SiN analysis. The ground part of the stimulus is based on tonal elements that are distributed randomly in frequency-time space. At a certain point in time, we constrain a certain number of elements to remain constant from one time frame to the next. When there are enough elements that are on for long enough subjects hear a figure that emerges from the ground stimulus.

In the original version of the stimulus (Teki et al., 2013) it is impossible to say whether a figure is present or not based on the distribution of tonal elements over frequency at a single time point. The perceptual mechanism therefore requires a basis that operates over time. One possibility is a local mechanism based on adaptation within the frequency bands that remain constant. An argument against this is the fact that detection of the figure increases as a similar function of the number of elements, irrespective of whether the time window is 25 or 50 ms: an adaptation mechanism would be expected to depend on the absolute duration of the figure. Further evidence against the adaptation model is provided in our original study based on manipulations of the stimulus including placing broadband noise in alternate time frames and using “ramped” stimuli containing a systematic change over time in the frequencies comprising the figure. The detection mechanism is robust to these manipulations. We have developed a model for the process based on a mechanism that “looks” at the activity in auditory cortical neurons tuned to different frequencies to seek coherence between their activity (Teki et al., 2013). We can derive a single metric corresponding to the coherence between all frequency bands that predicts psychophysical performance well. I will argue in the next section that this process first occurs in high-level auditory cortex.

Work on listeners without a history of hearing symptoms has demonstrated correlation between the detection of SiN in the form of sentences in noise and both audiometry and figure-ground analysis (Holmes and Griffiths, 2019). The subjects were “normal listeners” (defined in terms of the average hearing levels over frequency) but showed variable threshold increases in the high-frequency (4–8 kHz) range of the audiogram. We demonstrated a significant correlation between the high-frequency audiogram and SiN ability. A version of the original figure-detection task showed a weak correlation with SiN ability with marginal significance, and a version of the task that required discrimination of figures based on a feature (a temporal gap in the figure) showed a medium correlation that was significant (*r* = 0.32, *p* ≤ 0.01, *n* = 97). Essentially, subjects had to discriminate two intervals containing the same figure with and without a gap in the middle. Hierarchal regression demonstrated that the audiogram and figure discrimination tasks together accounted for approximately half of the explainable variance in SiN and that the audiogram and figure-ground tasks accounted for independent variance.

**FIGURE 2:**
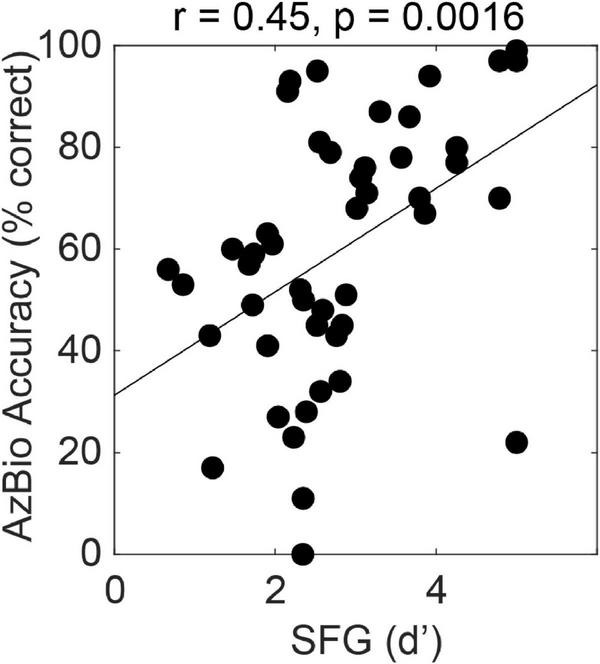
Correlation between performance for the figure ground task (SFG, stochastic figure ground) and a widely used US measure of sentence-in-noise perception, AzBio. The data are for 47 cochlear-implant users (see text).

The figure-ground task can also be used to assess cross-frequency grouping mechanisms in subjects with electrical hearing. Figure 2 shows the relationship between figure-ground detection and hearing sentences in noise in 47 subjects with cochlear implants. The implants were a mixture of conventional long electrodes that stimulate most of the cochlear partition and short electrodes that preserve low frequency acoustic hearing and stimulate the high-frequency basal region. We tested using a figure with components in the range above 1 kHz that was always in the electrical range even for users with the short devices. We see greater effect size (*r* = 0.45, *p* < 0.01, *n* = 47) for the relationship between figure-ground analysis and SiN compared to normal listeners, which is remarkable given that the figures are in a restricted range that does not include the whole speech range. Multiple linear regression demonstrated a significant effect of figure detection (standardised beta 0.29, *p* < 0.05) even after accounting for spectral modulation discrimination and temporal modulation detection as measures of cochlear function. A model containing all three of these non-linguistic factors accounted for 46% of the variance in SiN ability.

The tests of figure-ground analysis in both normal hearing and hearing-impaired listeners demonstrate a mechanism that explains variance in SiN ability independently of peripheral encoding of the stimulus. The idea is that a central grouping mechanism allows “pop out” or the formation of an auditory gestalt as a central process operating after peripheral analysis. The brain basis and clinical application of this work is considered below. This process is plausibly related to the perception of individual words in noise (although the experiments all measured correlations at the sentence-in-noise level). Mechanisms that contribute to the grouping together of words in sentences might also correlate with sentence-in-noise ability but not words in noise. The importance of grouping at this level was first suggested by a link between phonological working memory (WM) and SiN (Akeroyd, 2008; Dryden et al., 2017; Kim et al., 2020). From first principles, sentence comprehension has to require phonological WM at some point to allow the elements of the sentence to form the whole sentence. For sentences in noise, mechanisms might be based on separation of each word from noise followed by linking together of the elements by phonological WM, or linking together of the elements to form a “sentence gestalt” that is separate from the background noise. Correlation between phonological WM ability and SiN is better explained by the second mechanism. Debate about this correlation has centred on whether it holds in all listeners or whether age and hearing status moderate the relationship (Füllgrabe and Rosen, 2016). Moreover, there is ongoing discussion about the degree to which traditional phonological WM tasks depend on language skills (Schwering and MacDonald, 2020). We have been interested to develop non-linguistic paradigms to assess mechanisms that assess the grouping of acoustic elements over the timescale of sentences (seconds) that might contribute to SiN skill.

Recent work by my group examined the relationship between WM for non-verbal sounds and sentences in noise in a group of young listeners (Lad et al., 2020). The studies estimate WM capacity based on the precision with which non-speech sounds are held in memory. We use a delayed adjustment paradigm in which participants hear a sound, and then after a delay of several seconds adjust a second sound to match the sound heard in memory. The reciprocal of the standard deviation of the adjusted sound measures the precision of memory. In the context of distributed resource models of WM that have been applied to the visual and auditory domains (Bays and Husain, 2008; Kumar et al., 2013), this yields a measure of the resource available for WM: the greater the resource, the greater the precision. The study of Lad et al. (2020) showed a significant correlation between ability for sentences in noise (subjects had to match heard sentences to a matrix of written possibilities) and frequency precision (*r* = 0.36, *p* < 0.05, *n* = 44) but not amplitude-modulation-rate precision (where precision is defined for both measures based on the distribution of responses as above). No correlation was seen between the audiogram and the sentence-in-noise task. A possible interpretation of the dissociation between frequency and modulation precision is in terms of a critical importance of WM for the grouping of sources (like voices) that need to be yoked together in the foreground and are defined by stable frequency properties, as opposed to shorter events (like words). But further data on over 100 listeners has shown a significant correlation with WM for both frequency and amplitude-modulation rate with a moderate effect size (Meher Lad–unpublished observation). This suggests an alternative possibility–that there might be a common WM resource for storing acoustic features that is a determinant of SiN ability.

As an aside, the study of Lad et al. (2022) also examined links between musicality and acoustic WM and found a significant correlation. There is a longstanding debate about whether musicians have greater perceptual abilities relevant to music such as frequency discrimination: see (Moore et al., 2019) for a recent investigation and discussion. In a further study (Lad et al., 2022) the correlation between musicianship (based on the Goldsmith’s Musical Sophistication Index) and perceptual discrimination and WM for frequency was examined. The study showed a correlation with frequency WM but not perception of frequency. The data highlight an interesting specific link between musicality and WM for frequency for which a causal relationship could in principle be in either direction.

## Brain mechanisms for auditory cognition relevant to speech-in-noise perception

The behavioural experiments above suggest auditory cognitive bases for speech in noise at a pre-linguistic level relevant to words in noise (figure-ground analysis) and sentences in noise (WM for sound). These explain variance in SiN separate to that associated with cochlear measures. Linguistic factors represent another cause of variance in SiN. There are strong priors that implicate cortex in these mechanisms. In the case of figure-ground analysis a mechanism is required “looks” between widely separated frequencies that are, in general, represented in separate neurons in the ascending auditory pathway and primary auditory cortex. This suggests a mechanism in cortex beyond primary auditory cortex. In the case of WM for non-speech sounds a basis in auditory cortex or frontal cortex might be considered, based on early studies using musical stimuli (Zatorre et al., 1994).

Studies of the brain basis for figure-ground analysis have been based on a primate model that we have developed and human studies using functional magnetic resonance imaging (fMRI), magnetoencephalography (MEG) and invasive neurophysiology. The studies support a system based on high-level auditory cortex and parietal cortex.

Unlike SiN, studies of the underlying auditory cognitive bases for SiN that do not require linguistic processing can be studied in the macaque. Macaques have a similar frequency range for hearing to humans (Heffner and Heffner, 1986), and a similar lower limit of pitch (Joly et al., 2014). The auditory cortex is situated in the superior temporal plane at the top of the temporal lobe as in humans. The model allows systematic neurophysiology in a way that would never be possible in humans. Recordings of multiunit activity demonstrate tonic responses to figure onset that are present in all three auditory core areas in the superior temporal plane (Schneider et al., 2021). We can also carry out fMRI in the macaque to measure BOLD activity in neuronal ensembles: this allows a direct comparison with human studies. The macaque studies show activity associated with figure perception in high-level cortex over the lateral part of the superior temporal gyrus in the superior temporal lobe in the region of parabelt cortex, at a higher level in the auditory hierarchy than the core areas (Schneider et al., 2018).

Human fMRI also demonstrates BOLD activity over the lateral part of the superior temporal lobe corresponding to the presence of figures (Teki et al., 2011), in a human homolog of auditory parabelt. The human work also shows activity in the intraparietal sulcus. MEG has also demonstrated tonic response to figure onset arising from auditory cortex and intraparietal sulcus (Teki et al., 2016). Local-field-potential recordings from twelve neurosurgical candidates have demonstrated local-field potentials to figure onset that arise from early auditory cortex (human core homologs in the superior temporal plane) and high frequency oscillatory activity in the gamma band that arise from the lateral part of the superior temporal lobe (Gander et al., 2017).

Studies of the brain basis for acoustic WM analysis have been based on human studies using fMRI, and invasive neurophysiology. The studies support a system based on auditory cortex, inferior frontal cortex and the hippocampus. The studies have been based on a paradigm in which subjects hear two tones and are required to remember one after a retro-cue. After a delay of seconds, they are required to recall the tone.

fMRI has shown activity in auditory cortex during the memory maintenance period of this paradigm (Kumar et al., 2016). The activity was present in human core and belt homologs in superior temporal plane. This is not surprising but is not a given based on studies of visual WM using a similar retro-cue in which decoding of memory content from delay activity in visual cortex was possible but where activity levels did not increase (Harrison and Tong, 2009). I would also point out “activity silent” models for WM maintenance in which WM maintenance is based on synaptic strength rather than ongoing activity *per se* (e.g., Wolff et al., 2017). fMRI also demonstrated involvement of the inferior frontal cortex in WM maintenance (Kumar et al., 2016), which is also not surprising given the musical studies referred to above.

What was less anticipated in the fMRI WM study was the involvement of the hippocampus in WM maintenance (Kumar et al., 2016). The hippocampus is conventionally regarded as a part of the system for episodic rather than WM. We needed to use a long delay period in the fMRI study because of the sluggish BOLD response and one idea is that episodic measures might have been engaged during the BOLD experiment. But we have now carried out six sets of intracranial recordings on neurosurgical candidates with a much shorter delay and demonstrated consistent low-frequency oscillatory activity during WM maintenance in medial temporal lobe structures: hippocampus and parahippocampal gyrus (Kumar et al., 2021). Readers interested in a general account of how the computational machinery of the hippocampus might be used for auditory analysis are referred to Billig et al. (2022).

In summary, although fundamental auditory cognition relevant to SiN analysis need not explicitly engage the language system, the auditory-pattern analysis required engages cortical mechanism well beyond what is conventionally regarded as auditory cortex. I suggest that a complete account of the brain bases for speech in noise needs to consider auditory cognition in addition to higher-level linguistic analysis.

## Final comments: Possible clinical implications

I have developed an argument that problems with auditory cognitive mechanisms explain difficulties with speech in noise that cannot be accounted for by the audiogram. Defined in this way, auditory cognitive deficits might be considered a type of “hidden hearing loss.” The area is controversial. Hidden hearing loss is sometimes used as a synonym for cochlear synaptopathy (Kujawa and Liberman, 2009): the loss of synapses between inner hair cells and the afferent auditory nerve caused by noise exposure. Valderrama et al. (2022) consider other possible bases including auditory nerve demyelination and elevated central gain and mal-adaptation in brainstem auditory centres. Despite the controversy, the debate about bases for hidden hearing loss has consistently focussed on mechanisms in the ascending pathway. The cortical mechanisms I have described here add another level of complexity. Further work is required to examine the contribution of cortical figure-ground analysis and acoustic WM to SiN when both conventional measures of cochlear function and measures of hidden hearing loss due to brainstem factors are taken into account.

A major driver of this work is to develop new behavioural and brain tools that might allow better prediction of the potential success of hearing restoration using hearing aids or cochlear implants. The stimulus in Figure 1 might be thought of as an audiogram for acoustic scene analysis that might realistically be used alongside conventional pure tone and speech audiograms in the audiology clinic. We have developed simple brain measures of figure-ground analysis based on EEG that could also be used in any clinical centre (Guo et al., 2022).

Finally, understanding of auditory cognition relevant to speech in noise can potentially shed light on how hearing loss in middle life explains 9% of dementia cases (Livingston et al., 2017, 2020). We consider possible models in Griffiths et al. (2020), including the idea that this might be due to interaction between high-level mechanisms for auditory cognition beyond the auditory cortex, that are stressed by natural listening in subjects with hearing loss, and the pathological processes responsible for dementia. The idea that follows is that speech in noise and its auditory cognitive determinants, rather than simple hearing loss, is the critical determinant of dementia.

## Data availability statement

The original contributions presented in the study are included in the article/supplementary material, further inquiries can be directed to the corresponding author.

## Ethics statement

The studies involving human participants were reviewed and approved by the IRB, University of Iowa Hospitals and Clinics. The patients/participants provided their written informed consent to participate in this study. The animal study was reviewed and approved by Home Office Project Licence to A. Thiele.

## Author contributions

The author confirms being the sole contributor of this work and has approved it for publication.
